# Supplementary figures and images for: Automated black-box boundary value detection
Source: PeerJ Comput Sci. 2023 Nov 7;9:e1625. doi: 10.7717/peerj-cs.1625 (PMC10702978; doi:10.7717/peerj-cs.1625)

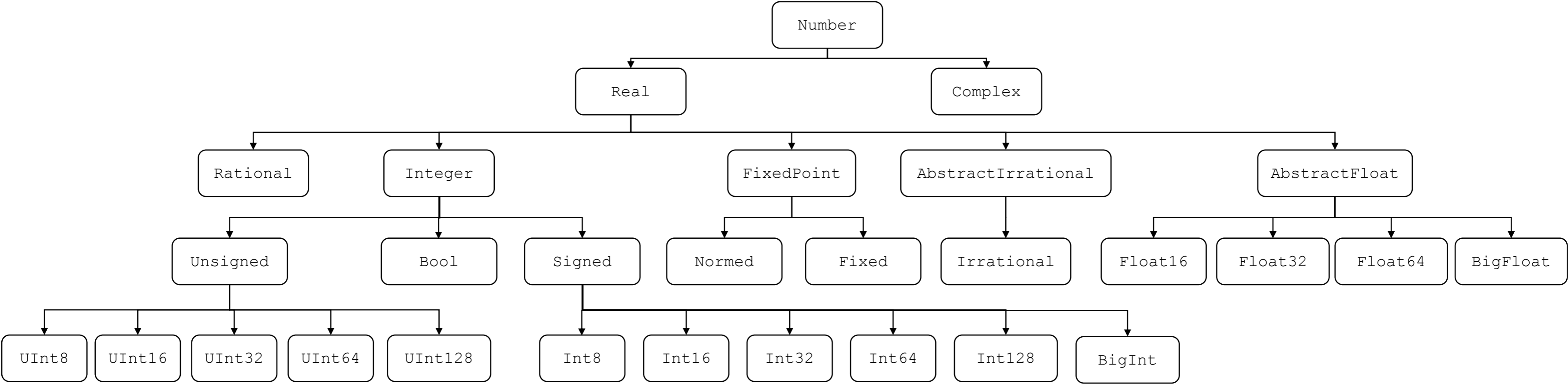

Supplement: Supplemental Information 1 [file peerj-cs-09-1625-s001.pdf]
